# Genetic Diversity of the Critically Endangered Lake Minnow *Eupallasella percnurus* in Poland and Its Implications for Conservation

**DOI:** 10.1371/journal.pone.0168191

**Published:** 2016-12-22

**Authors:** Dariusz Kaczmarczyk, Jacek Wolnicki

**Affiliations:** 1 Department of Environmental Biotechnology, University of Warmia and Mazury in Olsztyn, Słoneczna 45G, Olsztyn, Poland; 2 Pond Fishery Department, Inland Fisheries Institute, Główna 48, Żabieniec, Piaseczno, Poland; Sichuan University, CHINA

## Abstract

The lake minnow (*Eupallasella percnurus*) is critically endangered. In this paper we characterize the genetic properties of this fish over its range of occurrence in Poland and propose the use of this knowledge in its active protection. Twelve populations of lake minnow from across its range in Poland were investigated. 13 microsatellite loci were investigated to evaluate genetic variation and distance among populations. The magnitude of the genetic bottleneck or founder effects was investigated. In the studied populations, the allelic diversity and heterozygosity showed that genetic variation in this species is low. At most loci, only 2–3 alleles per population were detected. The average number of alleles detected across all loci was 35, and ranged from 24 to 53. The average observed heterozygosity (*H*_*o*_) across all investigated loci was 0.38 (range 0.21–0.59); the average expected heterozygosity (*H*_*e*_) was 0.36 (range 0.18–0.55). The populations remained in Hardy-Weinberg equilibrium. The average Garza-Williamson *M* index value for all populations was low (0.47), suggesting a reduction in genetic variation due to a founder effect or a genetic bottleneck. Genetic distance among populations was high or very high (F_ST_ range: 0.20–0.64; δμ^2^ range: 1.32–16.98); this was likely a consequence of low gene flow among isolated populations, a founder effect or other genetic bottleneck, and strong genetic drift. The large genetic differences among the investigated lake minnow populations are likely to also exist among other populations of this species, and knowledge of these differences should inform active protection programs based on translocation of wild or cultivated fish of this species. The method presented here can potentially be applied to any population of lake minnows or closely related species.

## Introduction

The lake minnow, *Eupallasella percnurus* (Pallas, 1814), is a tiny cyprinid fish species that is widely distributed in the Northern Hemisphere, from Poland in the west, throughout Northern Asia, and to the Pacific coast in the east [1], [2]. Although this species is not considered to be endangered globally [3], in Poland it is one of the rarest and the most imperiled freshwater fish species. This is due to the specificity of the lake minnow's habitats, which are very small and shallow water bodies primarily of anthropogenic origin, such as former peat or clay excavations. These water bodies have a limited period of existence (usually several decades) and are highly vulnerable to total destruction from natural causes (e.g., limited precipitation, shallowing) or human activity (swamp draining, filling in, littering).

Currently, lake minnows are believed to be present at about 160 sites in Poland, at least 75% of which are in danger of being destroyed [4]. For these reasons, the lake minnow is listed as critically endangered in the Polish Red Book of Endangered Animals. This species is not only under strict protection but also considered to require active protection measures, and it is a priority species within the European Ecological Natura 2000 Network [5].

Because genetic variation promotes adaptation to changing environmental conditions [6], such as those that threaten the lake minnow in Poland, information on the genetic variation in these lake minnow populations and the genetic differences between the populations is needed for an effective active-protection strategy. However, such knowledge is fragmentary because it is based on only one pair of populations [7] and one pilot-scale study [8].

To assess genetic variation for the management of genetic resources of many endangered fish species, polymorphic fragments of microsatellite DNA markers have been successfully used, e.g. [9], [10], [11]. Thus, the aim of this study was to use microsatellite polymorphisms to comprehensively characterize the genetic properties of lake minnow populations over the entire range of this species in Poland.

## Materials and Methods

### Fish samples

**Fig 1:**
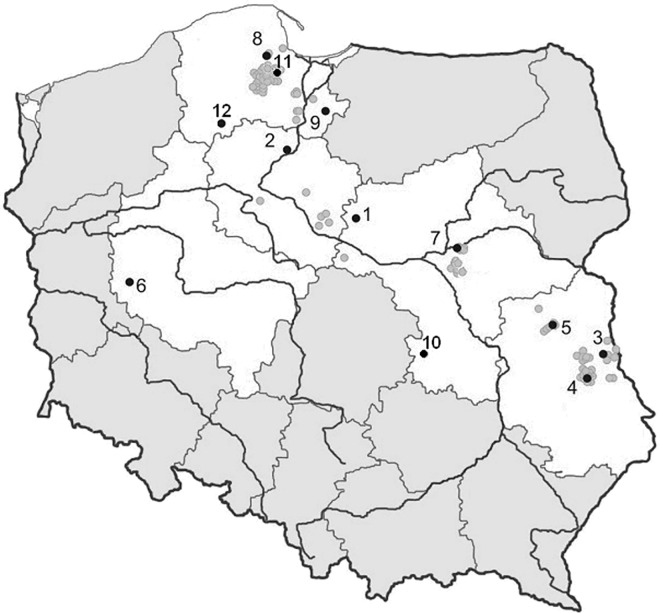
All sites of lake minnow occurrence in Poland (grey circles). Sites investigated in the present study (black circles): Sośniak (SO), Guzy (GU), Mikołajki Pomorskie (MP), Chojnice (CH), Sartowice (ST), Bledzewo (BL), Barłożnia Wolsztyńska (BW), Kowalicha (KO), Łojków (LO), Bełcząc (BE), Podpakule (PP), and Siedliszcze (SI). From each population, 48 fish were selected randomly, and samples were taken from them.

Adult fish from 12 lake minnow populations from throughout the species' entire range (Fig 1) were caught in baited traps [12].

To anesthetize the fish, they were placed in water that contained 0.45 g 2-phenoxyethanol /dm^3^. Material for genetic analyses was then taken by clipping approximately 20 mm^2^ from the ends of the fishes’ left pelvic fins; these fin fragments were collected from 48 fish from each population. Immediately after collection of these fragments, the fish were released into the same water bodies where they had been caught. All of these procedures were approved by the Ethics Commission in Olsztyn, Poland (permit number 22/2010). The wet samples were first placed on a 15 x 12 cm plate that was wrapped in foil, and then the samples were taken to an air-dryer for preservation. After drying each fin fragment was numbered, wrapped in new fragment of aluminum foil, and placed in separate 1.5 ml Eppendorf tube.

### DNA extraction

Genomic DNA was extracted and purified from fin tissues using a Sherlock AX DNA Extraction and Purification Kit, or a DNA and Genomic Mini AX Tissue SPIN DNA Extraction and Purification Kit. The extraction procedure was performed following the manufacturer’s recommendations (A&A Biotechnology). DNA samples were stored at a temperature of –20°C. The integrity of the DNA samples was visually inspected following electrophoresis in a 1.5% agarose gel stained with etidium bromide. All agarose gels were photographed using a gel imaging system and the images were digitally recorded. Samples of the DNA yields were quantified by spectrophotometric analysis; only samples containing more than 30 pg/μl of double-stranded DNA qualified for the PCR stage.

### PCR amplification

The assessment of genetic variation was based on 13 polymorphic microsatellites. The primer sequences used for amplification of DNA fragments, including microsatellites *Ca3 Ca4*, and *Ca12*, were taken from [13] and [14]. The primer sequences used for amplification of loci *Z9878*, *Z10362*, and *Z13419* were taken from GeneBank. Microsatellites *Eupe1*–*Eupe9* were amplified using primer sequences described by Kaczmarczyk and Gadomski [15] (http://www.ncbi.nlm.nih.gov). The primer sequences, repeat motifs, and their accession numbers are given in Table 1.

**Table 1:** The primers sequences, repeat motifs and gene bank accession numbers of the investigated microsatellite fragments

Locus	Motif	Primer sequence	GenBank accession number
*Z9878*	CA	F: ACATCCACACCGTCTGTCAA	G39785
		R: CACGTCATCAAGCAGAGGAA	
*Z10362*	CA	F: GGTGACCTCATGGAAGCATT	G40857
		R: AGCTACTGAAACCCTTTGGC	
*Z13419*	TG, TC	F: AGGTTTCAGAGCCCTCATCA	G41762
		R: CATGTGAACTCTGAAGCCCA	
*Ca3*	TAGA	F: GGACAGTGAGGGACGCAGAC	AF277575
		R: CCGTAAAATTTGGGGGCTAGA	
*Ca4*	AC	F: CGGTATCGGTGCATCCCTAAA	AF277576
		R: AACAGCGCGAGCGTCATTC	
*Ca12*	TAGA, CAGA	F: GTGAAGCATGGCATAGCACA	AF277584
		R: CAGGAAAGTGCCAGCATACAC	
*Eupe1*	GGAT	F: TAAACCAATAAAATCTATCAAATGTGG	JQ937252
		R: TGCTATTTAGTTTGTTTAAATGTAGCA	
*Eupe2*	ATC	F: AAGGCTGGTGACTTTCCAGA	JQ937253
		R: CTGGTCAGCTGAAGCATTTG	
*Eupe4*	ATCT	F: TCAGACAGACACTGCAACGA	JQ937245
		R: CTGCTGTGTTCTGGTGATGC	
*Eupe5*	GT	F: GCATTCAGTGCATGGAGGAG	JQ937246
		R: TCATCTCTAAATGCCCTCGC	
*Eupe6*	CCAT	F: GCCATGGGATTTTAATGACC	JQ937247
		R: GGCCATGAACAACCTGCTAA	
*Eupe7*	CA	F: TTCACTAACAGGCCATGCAA	JQ937248
		R: CATGACCAACGACTGACACTG	
*Eupe9*	TCCA	F: CTAGCTGTCAGTCCATCCATC	JQ937250
		R: CGATAGAGATGAGAAGGCAACAC	

The PCR amplification was performed in a 30 μl reaction. The forward primer of each primer pair was 5’ end labeled with fluorescent dyes (6FAM, VIC, NED, and PET). The PCR reaction was started with denaturation of the DNA at a temperature of 95°C for 3 minutes. Next, 34 cycles were performed: each cycle consisted of denaturation (1 minute at 95°C), annealing (30-45s at 52–65°C; a different time and temperature were used for each primer set), and elongation at 72°C for 35-45s, depending on the specific microsatellite. After the last cycle, the final elongation was performed for 15 minutes at 72°C. The PCR product was verified by electrophoresis in a 2% agarose gel and stained using ethidium bromide.

### Genotyping

Each microsatellite marker was amplified independently of the others, but for genotyping they were arranged in five sets (Table 2).The lengths of the amplified DNA fragments were determined using an Applied Biosystems 3130 Genetic Analyser with GS400LIZ size standards.

**Table 2:** Microsatellites used in multiplex genotyping assays

Assay name	Name of microsatellite fragment and phosphoramide dye			
	6-FAM	VIC	NED	PET
I	*Z13419*	*Z10362*	*Z9878*	
II	*Ca3*	*Ca12*	*Ca4*	Eupe7*
III	*Eupe9*			*Eupe6*
IV	*Eupe1*		Eupe5**	
V	*Eupe4*	*Eupe2*	Eupe5**	Eupe7*

* Marker *Eupe7* was genotyped in assay II or V

** Marker *Eupe5* was genotyped in assay IV or V

Within these sets, the products of each individual microsatellite amplification were marked with different attached phosphoramide labels, thus enabling their separation in multiplex mode. Fragment size and allele determination were performed using GeneMapper 3.0 software (Applied Biosystems), following the manufacturer’s recommendations.

### Statistical analyses

The tetrasomic locus *Ca4* was divided into two isoloci, *Ca4A* and *Ca4B*, in order to accommodate the requirements of MSA and Arlequin software. Genetic variability was measured using observed heterozygosity (*H*_*o*_), expected heterozygosity (*H*_*e*_) [16], the average number of alleles, and allelic frequency. The number of alleles observed per locus, allele frequency, allelic range, and allelic richness were computed by MSA software [17].

The observed heterozygosity (*H*_*o*_) was calculated for each locus using an algorithm described by [16].

The Exact Hardy Weinberg (H-W) test [18] was used to test for deviations from H-W equilibrium. The test was performed separately for each locus in each population as well for all loci in the given populations. The number of steps in the Markov chain equalled 1,000,000 and the number of dememorization steps equalled 100,000. The deviations were considered significant if *p* ≤ 0.05.

Observed heterozygosity (*H*_*o*_) and expected heterozygosity (*H*_*e*_) were calculated using Arlequin 3.0 software [19].

The likely occurrence of a bottleneck or founder effects, and its influence on within-population genetic variability was based on the Garza-Williamson *M* index (the number of alleles divided by the allelic range). This index [20], including Excoffier’s adjustment, was calculated using Arlequin 3.0 software [19]. The index is based on the observation that in bottlenecked populations the number of alleles observed in an allelic range is less prone to be reduced than the allelic range itself (this is because the allelic range is the difference in the number of nucleotides of the longest and of the shortest alleles of a microsatellite fragment divided by length of the microsatellite’s repeat motif, so unless the longest or shortest allele is changed, the allelic range will not be changed). The average value of the *M* index calculated across all microsatellite markers ranged from 0.8 to 1, which is typical of populations in which genetic variation has not been reduced because of a bottleneck. *M* values from 0.6 to 0.8 indicate the possibility of a founder and/or bottleneck effects, and values lower than 0.6 indicate a strong founder and/or bottleneck effects.

Genetic divergence between populations was analysed using two different methods: the inbreeding coefficient (F_ST_) [21] and the variation in average allelic size (δμ^2^) [22]. F_ST_ values and their statistical significance were calculated with Arlequin 3.0 software [19]. The size of the genetic distance based on F_ST_ values and their ranges was interpreted according to [23] and [24]. Higher values of this coefficient (closer 1.0) and statistical significance indicate larger genetic differences between pairs of populations, whereas lower values (closer to 0.0) and a lack of statistical significance indicate genetic similarity. Genetic divergence was also estimated by using the sample-size independent δμ^2^ -method (calculated with MSA software [17]); with this method, higher values indicate larger genetic differences between populations and smaller values indicate small differences.

The significance of Pearson correlations between geographical distance and genetic distances between populations were tested with a two-tailed Mantel test. Geographical distance (km) was measured in a straight line between the water bodies inhabited by the investigated populations, and genetic distance was estimated with the F_ST_ and δμ^2^ methods. Significance was set at *α* = 0.05, and 100,000 permutations were performed, using Version 2015.2.02.18165 of the XLSTAT add-on to MS Excel 2003 (Microsoft, USA). The same method was used to test the correlation between the genetic distances estimated using the F_ST_ and δμ^2^ methods.

Genetic divergence between the investigated populations and genetic differences within populations were also analyzed using the assignment test. The log likelihood of the occurrence of given fish genotypes in their source or alternative population was calculated using Arlequin 3.0 software. The obtained likelihood values were presented as points in a system of coordinates following [25].

To verify the results of the assignment test we performed a Bayesian analysis of population structure with STRUCTURE 2.3.4 software [26]. This analysis focused on inferring the possibility that subpopulations exist within the investigated populations, and that there are admixtures of populations, and on assigning fish to their source population or other populations. To test the K parameter, it was increased stepwise from 12 (the number of populations) to 20 (12 populations plus 8 suspected subpopulations). The Burnin Period was 100000, and the number of MCMC reps after Burnin was also 100000.

The contribution of the specific components of genetic variance to the total variance observed among all 12 investigated populations was estimated by means of hierarchical analysis of molecular variance (AMOVA) [27]. These calculations were performed using Arlequin 3.0 software [19] with 1000 permutations. The threshold for significance was set at *p* = 0.05.

## Results

### Intrapopulation genetic diversity

#### Allelic diversity

All of the 13 microsatellites in this study were successfully amplified. Across the investigated markers, 96 alleles were detected. All investigated microsatellites were polymorphic in at least one population, but the polymorphisms differed between populations. Several microsatellites were monomorphic in one or more populations. Only a few microsatellites had greater than six alleles (Table 2). At the population level, the degree of polymorphism across all investigated loci was low (on average, 35 alleles per population). All populations differed in the number of alleles detected at a given locus as well as the overall number of alleles identified across all investigated loci. The total number of alleles ranged from 24 in population CH to 53 in population BE. The allelic richness was low and varied from 1.85 alleles per locus in population CH to 4.08 in population BE (Table 3).

**Table 3:** Genetic properties of investigated populations. Number of alleles detected at investigated loci, total number of alleles (*N*), allelic diversity (*AD*), observed (*H*_*o*_) and expected (*H*_*e*_) heterozygosity calculated across all loci in investigated populations, value of Garza-Williamson index (*M*). For populations, see Fig 1.

Locus	Population											
	BL	KO	SI	SO	MP	PP	BE	ST	BW	LO	CH	GU
*Z9878*	2	2	2	2	2	2	2	2	2	2	2	2
*Z10362*	2	2	2	3	2	3	2	2	2	2	1	2
*Z13419*	2	2	2	2	2	2	1	1	2	3	2	2
*Ca3*	2	3	1	1	1	2	8	1	1	3	1	2
*Ca4*	3	3	2	4	3	3	4	3	4	4	3	3
*Ca12*	5	8	4	5	1	6	9	8	6	7	4	8
*Eupe1*	4	6	1	6	1	7	7	2	1	1	3	3
*Eupe2*	5	4	2	1	1	1	1	4	2	5	1	1
*Eupe4*	2	2	2	2	2	2	3	1	2	1	2	2
*Eupe5*	1	3	1	1	1	2	1	2	1	1	1	1
*Eupe6*	2	3	1	2	1	4	3	4	3	5	2	4
*Eupe7*	2	3	1	2	2	5	6	4	1	3	1	2
*Eupe9*	2	4	6	3	4	5	6	2	4	4	1	1
*N*	34	45	27	34	23	44	53	36	31	41	24	33
*AD*	2.62	3.46	2.08	2.62	1.77	3.38	4.08	2.77	2.38	3.15	1.85	2.54
*H*_*o*_	0.25	0.55	0.35	0.41	0.21	0.59	0.47	0.34	0.37	0.44	0.22	0.39
*H*_*e*_	0.25	0.50	0.29	0.35	0.18	0.55	0.47	0.30	0.35	0.46	0.24	0.37
*M*	0.56	0.65	0.38	0.51	0.31	0.58	0.48	0.43	0.44	0.42	0.40	0.42

#### Heterozygosity and Hardy-Weinberg equilibrium

In order to evaluate population-wide genetic variation, the observed (*H*_*o*_) and expected (*H*_*e*_) heterozygosity was calculated (Table 3). In general, genetic variation was low, with average values for observed and expected heterozygosity of 0.38 and 0.36, respectively. The values of *H*_*o*_ and *H*_*e*_ for individual populations varied considerably. In populations PP and KO, these values exceeded 0.50, but in three others (BL, MI, and CH), the values were lower than 0.30 (Table 3). In all populations, the mean *H*_*o*_ value was close to the average percentage of heterozygotes (*H*_*e*_) expected at H-W equilibrium. When calculated across all markers, departures from this equilibrium were not significant (*p* > 0.05). Departures were only found at the level of individual loci (Table 4). Departures at locus *Z9878* were significant in most populations, but significant deviations at other loci were detected only in some populations.

**Table 4:** Results of the test for deviation from Hardy-Weinberg equilibrium. For population names, see Fig 1.

Locus	Population											
	BL	KO	SI	SO	MP	PP	BE	ST	BW	LO	CH	GU
*Z9878*	*	*	*	*	*	*	*	*	*	*	-	*
*Z10362*	-	-	-	-	-	-	*	-	-	-	*mn*	-
*Z13419*	-	-	*	-	-	-	*mn*	*mn*	*	*	-	*
*Ca3*	-	-	*mn*	*mn*	*mn*	-	-	*mn*	*mn*	-	*mn*	-
*Ca4*	-	-	-	-	-	-	-	-	-	-	-	-
*Ca12*	-	-	-	-	*	*	*	-	*	-	*	*
*Eupe1*	*	-	-	-	*mn*	-	-	-	-	*mn*	-	-
*Eupe2*	*	*	*	*mn*	*mn*	*mn*	*mn*	-	-	*	*mn*	*mn*
*Eupe4*	-	*	*	*	-	-	-	*mn*	-	*mn*	*	-
*Eupe5*	*mn*	-	*mn*	*mn*	*mn*	-	*mn*	-	*mn*	*mn*		*mn*
*Eupe6*	-	-	*mn*	-	-	-	-	-	*	-	-	-
*Eupe7*	-	-	*mn*	*	-	*	-	-	*mn*	-	*mn*	-
*Eupe9*	-	-	*	-	-	*	-	-	-	-	*mn*	*mn*

*(mn)*–monomorphic locus

(-)–no deviation or no significant deviation

(*)–significant deviation, *p* ≤ 0.05

#### Bottleneck and founder effects

The average Garza-Williamson *M* index value across the investigated populations was 0.47. This value is lower than 0.60, which indicates that founder and/or bottleneck effects had a great impact on genetic variations in these populations (Table 3). The *M* value was highest in population KO (0.65), which is the only population where the *M* index was greater than 0.60. This indicates that, in this newly established population, the founder/bottleneck effect was relatively small. The lowest *M* value was in population MP (0.31), which suggests a sustainable reduction of genetic variation in this population as a result of founder and/or bottleneck effects.

### Genetic structure of populations and likelihood of admixture

**Fig 2:**
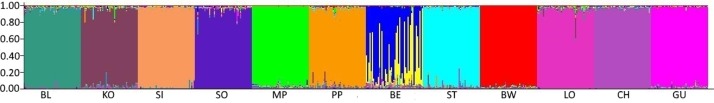
Bar plot of Bayesian analysis of population structure (K = 14) showing the proportion of individual fish’s membership that was assigned to their source populations and to other populations, and the probability of admixture with other populations.

Bayesian analysis of the population structure (K = 12–20) did not show the existence of any subpopulations within the investigated populations. In Fig 2 (abbreviations are defined in Fig 1), each vertical line represents the inferred proportion of an individual fish’s membership in each population. In 11 out of the 12 populations, the proportion of membership in the fishes’ source populations is much higher than that assigned to alternative populations. In the BE population, the long yellow and blue vertical lines indicate that there is substantial admixture of this population with fish from another population or populations. Moreover, the BE population is the only one in which a trace of admixtures was observed in almost the whole range of K values from K = 13 to K = 20. In other populations, the traces of admixture were not clear, because in some of them these traces started at K15 or K16, then disappeared at higher K values. At the highest K values (18–20), the analysis showed a high similarity between fish from very distant populations, and admixture between these populations, indicating that the results at these values were misleading.

### Genetic distance between populations

Based on F_ST_ values, the genetic distances between most populations were very large (Table 5), and all genetic differences between populations were significant at *p* < 0.001. The largest genetic distance (F_ST_ of 0.71) was found between populations MP and SI, and there were also very large distances between population BW and all other populations except LO. The smallest genetic distances were found between populations KO and BE (0.22), KO and PP or LO (both 0.24), BE and SO (0.27), and BE and PP (0.29). The average genetic distance that was estimated using F_ST_ values was 0.44 (SD 0.11; normal distribution).

**Table 5:** Genetic differences between populations based on δμ^2^ (normal font) and F_ST_ (italic font) values. For population names, see Fig 1.

	BL	KO	SI	SO	MP	PP	BE	ST	BW	LO	CH	GU
**BL**	-	2.42	16.42	6.54	3.56	2.31	8.05	2.35	12.52	5.16	4.82	6.25
**KO**	*0*.*33*	-	10.63	5.73	2.72	3.56	2.56	1.14	10.44	3.14	3.03	3.68
**SI**	*0*.*56*	*0*.*47*	-	13.82	14.20	13.05	8.45	10.21	16.98	13.82	12.75	10.04
**SO**	*0*.*59*	*0*.*38*	*0*.*57*	-	5.62	7.33	6.95	5.12	8.23	6.06	2.54	1.33
**MP**	*0*.*38*	*0*.*39*	*0*.*71*	*0*.*64*	-	5.24	7.44	1.60	11.65	6.12	2.13	4.26
**PP**	*0*.*42*	*0*.*24*	*0*.*45*	*0*.*41*	*0*.*43*	-	8.86	3.93	11.54	6.78	7.88	8.17
**BE**	*0*.*39*	*0*.*22*	*0*.*37*	*0*.*27*	*0*.*49*	*0*.*29*	-	4.66	12.16	4.46	5.29	3.91
**ST**	*0*.*39*	*0*.*31*	*0*.*53*	*0*.*51*	*0*.*49*	*0*.*38*	*0*.*34*	-	10.96	4.51	2.50	3.05
**BW**	*0*.*60*	*0*.*40*	*0*.*60*	*0*.*46*	*0*.*60*	*0*.*45*	*0*.*44*	*0*.*55*	-	3.81	9.58	9.76
**LO**	*0*.*49*	*0*.*24*	*0*.*53*	*0*.*42*	*0*.*52*	*0*.*42*	*0*.*32*	*0*.*45*	*0*.*32*	-	4.60	5.36
**CH**	*0*.*51*	*0*.*38*	*0*.*61*	*0*.*53*	*0*.*48*	*0*.*47*	*0*.*41*	*0*.*32*	*0*.*55*	*0*.*47*	-	1.46
**GU**	*0*.*52*	*0*.*30*	*0*.*53*	*0*.*35*	*0*.*51*	*0*.*42*	*0*.*27*	*0*.*38*	*0*.*42*	*0*.*32*	*0*.*31*	-

The magnitudes of the genetic distances between populations were also estimated using the δμ^2^ method (Table 5). This method confirmed that genetic distances between most of the populations were very large, although some of the specific results differed from those reported above. Genetic distances between population BW and other populations were generally large, with a maximum δμ^2^ of 16.98 between BW and SI. This method also uncovered a large genetic distance between populations SI and MP (14.20), SI and LO (13.82), and SI and SO (13.82). The smallest genetic distances (δμ^2^ < 2.0) were found between four pairs of populations: KO and ST (1.14), SO and GU (1.33), CH and GU (1.46), and MP and ST (1.60). The average genetic distance estimated by this method was 6.75 (SD 4.01; normal distribution). The values of genetic distance estimated by the F_ST_ method correlated with those estimated by the δμ^2^ methods (r(AB) = 0.534, p < 0.0001).

### Genetic distance versus geographical distance

The geographical distances separating the lake minnow populations in the present study differ considerably, and population BW is the most isolated from all the others (Fig 1). The shortest distance between this population and others is approximately 200 km (CH, ST, and BL); the largest distances between populations (500 km) are between BW and BE, and between PP and SI. The geographical distance between the genetically most similar pairs of populations (based on δμ^2^) ranged from a minimum of 25 km (SO and GU) to a maximum of 200 km (ST and KO).

In order to determine whether genetic distance was correlated with geographical distance between all pairs of populations, a two-tailed Mantel test was performed. When genetic distance was estimated with F_ST_ values, the correlation was small and not significant (r(AB) = 0.242, *p* > 0.05). When this distance was estimated with δμ^2^ values, the correlation was moderate and significant (r(AB) = 0.424, *p* < 0.05).

### Within population versus interpopulation genetic differences

**Fig 3:**
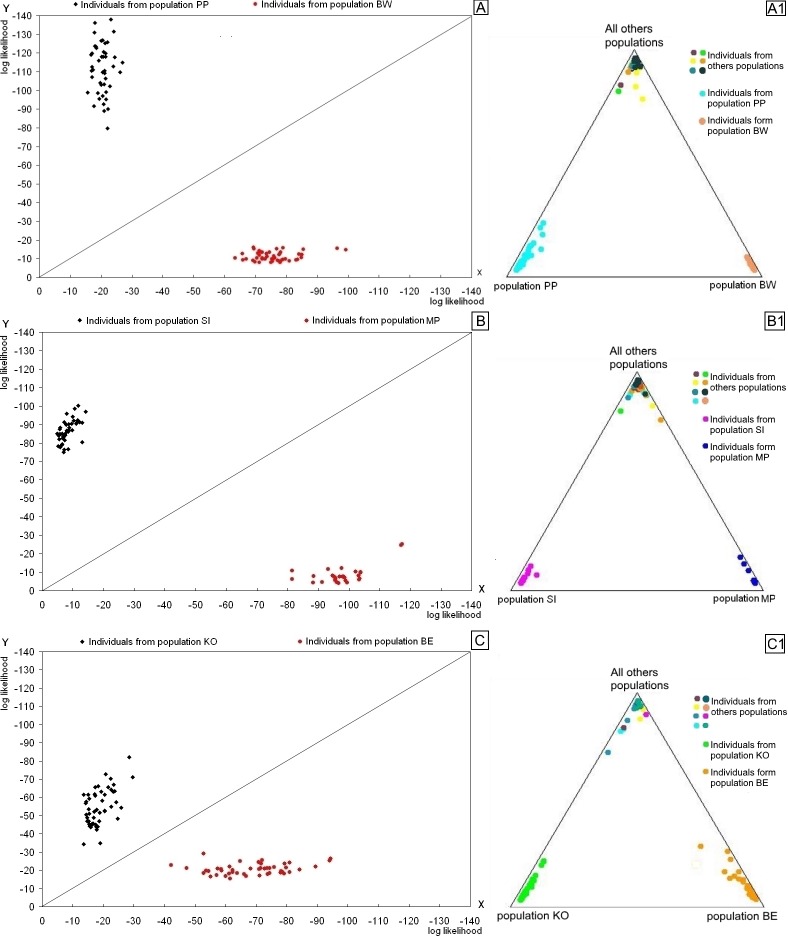
Results of the genetic assignment test and Bayesian analysis performed on pairs of populations. In graphs A, B, and C, points represent individual genotypes, and the axes show the log likelihood of the occurrence of a genotype in one population or the other. The triangle plots (A1, B1, C1) show the probability of the occurrence of fish genotypes in their source population or in an alternative population (lower left and right corners), as well as in all other investigated populations (upper corner). For population names, see Fig 1.

The results of the genetic assignment test showed that all fish genotypes had a higher likelihood of occurrence in their source populations than in alternative ones. Fig 3 shows examples of pairs of populations that are moderately diverse and have a large genetic distance between them (populations PP and BW, Fig 3A and 3A1), pairs that have low genetic diversity and a very large distance between them (SI and MP, Fig 3B and 3B1), and the pair that has the greatest diversity of all the investigated populations and a relatively small distance between them (KO and BE, Fig 3A and 3A1). The genetic assignment test confirmed the magnitude of the genetic variation observed within populations, as well as the genetic divergence between them. The triangle plots showing the results of the Bayesian analysis confirmed the results of the genetic assignment test in terms of within population genetic differences (the spread of the points in the plot), and in terms of genetic differences between populations (the distance between a cluster of points corresponding to a given population and to those corresponding to other populations). The Bayesian analysis also showed that individuals with a specific genotype have a much higher probability of occurrence in their source populations than in other populations. Moreover, the overlapping points in the upper corner of the triangle plots, which represent individuals from all other populations, confirm the differences between the two populations compared in the plot and all others.

In Fig 3, the distance between clusters of points reflects the distance between populations, and the spread of the points within a cluster reflects the genetic differences within the population. Graphs (A, A1) and (C, C1) show that in more genetically diverse populations, such as KO, PP, and BE, the points representing individual genotypes are spread over a relatively wide area above or below the diagonal. In populations with low genetic variation (such as MP), the points are grouped close to each other (SI) or overlap (MP) (Fig 3B and 3B1). In pairs of populations with large genetic differences (PP and BW, SI and MP), the points are further from the diagonal (Fig 3A and 3B) than in pairs of populations that are relatively similar to each other (Fig 3C).

Hierarchical analysis of molecular variance (AMOVA) showed that the percentage of variance within populations (Vb) was the smallest contributor to total molecular variation, and was not significant (*p* = 1) (Table 6). Most of the genetic variation was attributed to differences among populations (Va) and differences among investigated individuals (Vc). Both classes of components of genetic variance were significant (*p*<0.0001).

**Table 6:** The results of hierarchical analysis of molecular variance (AMOVA) showing the contribution the components to total genetic variation in the investigated samples. *p*<0.05 indicates significance.

Source of variation	d.f	sum of squares	Variance components	precentage of variation	*p* value
Among populations	11	1719.50	1.58 Va	44.36	<0.0001
Within populations	574	1014.34	-0.22 Vb	-6.11	1.0
Within individuals	586	1291	2.20 Vc	61.75	<0.0001
Total	1171	4024.83	3.57	100.0	

## Discussion

Our study is the first known scientific report that demonstrates a complete and validated method for evaluating the genetic variation and similarity within and between lake minnow populations. The method presented here uses the polymorphism of microsatellite DNA markers as a precise tool for evaluation of the genetic differences between individuals as well as between populations and stocks [27], [10], [28]. This method could potentially be used around the world with *Eupallasella percnurus* and closely related species. The results of this work provide information on the genetic diversity of the Polish populations of the lake minnow, and they provide valuable information that can be used for the active protection of this species within Poland and perhaps elsewhere.

In Poland, the most common lake minnow habitats are old (50–100 years) peat excavations, which have shallowed to less than 1 m at their deepest point because of intensive plant succession [5]. This feature means that lake minnow habitats can see a dramatic decline in water depth during droughts, which can reduce a population to only a dozen or so individuals. In fact, such situations have often been seen in the field [29]. However, the lake minnow has an exceptional ability to regenerate its populations from only a few individuals or to successfully initiate completely new populations with a low number of fish.

This combination of habitat vulnerability to drought and species regenerative ability suggests that lake minnow populations in Poland are frequently subject to founder effects and/or strong genetic bottlenecks, and the results of the present study are in agreement with this hypothesis, as shown by the measurements of heterozygosity and allelic diversity, the analysis of molecular variance and the assignment test [25]. It should also be stressed that the values of the Garza-Williamson index (*M*) for all the investigated lake minnow populations were very low, which indicates that all of them experienced severe genetic bottlenecks and/or founder effects. Knowledge of these values makes it possible to compare the probable reductions in population size in lake minnow populations to those in other species of finfish, reptiles, and mammals reported by [20] and [30]. An *M* index value lower than 0.8 shows a reduction in genetic variation in a population due to these effects, whereas an *M* value lower than 0.68 indicates a recent reduction in population size. It should be pointed out that in all populations in the present study, *M* indexes calculated across all markers were lower than 0.68 and sometimes did not reach even half of this.

Bottlenecks and founder effects reduce the effective size of a population and increase the rate of inbreeding. These effects usually reduce the genetic variation in a population [31], [32] and increase the rate of genetic differentiation between populations [33]. A reduction in the genetic variation in a population can be observed as a decrease in heterozygosity and allelic diversity [34]. In the lake minnow populations studied here, the values of *H*_*o*_ and *H*_*e*_ were low or very low in all populations, both on the level of a single locus and on the population level. It is noteworthy that, for example, *H*_*o*_ and *H*_*e*_ values for *Ca3* or *Ca4* were lower than those recorded in other fish species, such as *Campostoma anomalum* [13]. When the data were compared across all investigated loci, the values for the lake minnow were lower than those found in populations of fish like *Notropis mekistocholas* [35] or *Anaecypris hispanica* [36]. The average observed heterozygosity detected across all populations investigated in this study is almost the same as in the populations of the Australian lungfish *Neoceratodus forsteri* (*H*_*o*_ = 0.39) reported by [37]. The similarly low genetic variations in the populations of these species [37] confirm that the bottleneck effect can substantially reduce genetic variation.

In the present study, population BE was found to have the highest number of alleles and one of the highest levels of heterozygosity of all the investigated populations. The differences in the genotypes of individuals in this population indicate that admixtures with other populations took place. These admixtures could have occurred because this population is located in the upper part of the Tyśmienica River valley with several other lake minnow populations only 1–2 km away. Thus, the relatively high heterozygosity and allelic diversity of population BE suggests that local gene flow takes place among all the nearby lake minnow populations during the flood events that occur there from time to time. Such a situation is not experienced by any of the other 11 populations in the present study.

*H*_*o*_ and *H*_*e*_ values differed significantly at some markers but not at others. Although average *H*_*o*_ is close to *H*_*e*_ it is not clear if all populations remain at H-W equilibrium. Departures from H-W equilibrium at locus *Z9878* are known to occur in the genomes of cyprinid species [14], although the reason is unknown. Departures from H-W equilibrium at other loci may be the result of founder and/or bottleneck effects followed by a high rate of inbreeding.

Although there was little variation within the populations of lake minnow investigated in the present study, genetic variation between these populations was large, and it seems likely that the situation is similar in the majority of all lake minnow populations in Poland.

Although this conclusion differs from the results obtained by [8], those authors used a less sensitive method (analyses of mtDNA D-loop and rDNA ITS1 sequences) and limited amounts of biological material. Thus, our present findings should not be considered surprising, and they are in general agreement with the observations of [7]. The large interpopulation genetic differences that we observed are easily explained by the geographical isolation of the vast majority of the sites in which Polish lake minnows are found [5]. These genetic differences seem to result primarily from very low gene flow between populations. It is worthy of note that, due to its small size (mostly <10 cm total length) and relatively small population (0.5–1.5 thousand fish aged 1 year or more [12]), the lake minnow in Poland has never been of any commercial value nor has it been an attractive target for anglers. All of these factors have allowed this fish to avoid translocation over longer distances, both within and out of its natural range of occurrence in the country. Moreover, this species does not show any ability or inclination to migrate. All of these facts lead to the conclusion that gene flow is low, and it is generally possible only on a local scale, between closely situated populations such as BE and others that are close. People and floods are the most important vectors. In areas where many waterbodies inhabited by the lake minnow are in close proximity, the local populations can be described as a metapopulation consisting of a group of more or less geographically isolated subpopulations [38, 39]. In this situation, local extinctions and recolonization are typical, and some genetic interaction between the subpopulations can take place.

The analysis of molecular variance shows that the genetic differences between lake minnow populations in the present study is the major component of genetic variation in all investigated samples. In general, the genetic differences between pairs of populations are very large. In many cases, the magnitude of the genetic distance calculated using the F_ST_ method was comparable to or even larger than those of geographically distanced or isolated populations of sturgeons [40]. The magnitudes of genetic distances estimated by the δμ^2^ method were comparable to the values of bears inhabiting various regions of North America [41]. In many cases, genetic distances estimated using the δμ^2^ method between lake minnow populations were higher than those of other endangered animal species, such as the natterjack toad *Bufo calamita*, which inhabits various regions of Great Britain [42], or Mongolian and Norwegian horses [43].

Generally, the distances estimated by the F_ST_ and δμ^2^ methods were similar. The Mantel test showed that between all pairs of lake minnow populations, the results obtained for genetic distance using the two methods were in close agreement. Moreover, the sizes of pairwise within-population and interpopulation genetic differences were found to confirm the results of the genetic assessment test. We did not detect a clear correlation between geographic distance and the size of genetic differences existing between pairs of populations.

The results of the Mantel test suggest that the geographical distance between lake minnow populations may have some effect on genetic distance, but it is definitely not the key factor determining the extent of genetic differences.

### Implications for active protection

As was indicated above, the Polish lake minnow likely still inhabits its natural range of occurrence in the country. This commonly accepted view is also supported by the comparison of currently extinct lake minnow sites (approximately 100; [44] and currently existing sites (approximately 160; [5]. Thus, the large genetic differences between lake minnow populations that were observed in the present study should be considered a important feature of the Polish lake minnow in its natural state.

Active protection programs should be designed to preserve these genetic differences between populations. So far, active protection of lake minnow populations in Poland has been limited to the vicinity of Warsaw, in the central part of the country, where only a few sites of this species have survived until today. This long-term program, including captive breeding and translocations of cultivated juvenile fish (offspring of parents from a strong local population) was begun in 2002 [45]. The program has not been in existence long enough to comprehensively evaluate its efficiency. However, taking into account only the size and structure of populations, at least 5 out of 10 attempts undertaken in the years 2002–2009 seem to have been successful [46]. Activities of this kind obviously stimulate gene flow, but only on a local scale. It can be expected then, that they will not disturb the natural genetic variability of lake minnow populations over the whole range of this species in Poland.

The method developed and applied in this study has the potential to help maintain the genetic variation of lake minnow populations. The set of molecular markers used in this study enable identification a group of males and females within given population that are most genetically different from each other. These individuals can be assembled in spawning pairs using Genassemblage software [47] to produce highly genetically diverse juveniles in aquaculture conditions. Another method for conserving the genetic variation of this species is cryopreservation of sperm samples and genetic profiling to construct a bank of cryopreserved sperm taken from males inhabiting various populations in Poland. The genetic profiles of the sperm enable genetic resources to be managed. This method can be especially valuable for conservation of the populations that are single, most fragile to environmental changes and strictly isolated from another such as BW population, as is now done by our team.
