# Correction: Genetic Diversity of the Critically Endangered Lake Minnow *Eupallasella percnurus* in Poland and Its Implications for Conservation

**DOI:** 10.1371/journal.pone.0173012

**Published:** 2017-02-22

**Authors:** Dariusz Kaczmarczyk, Jacek Wolnicki

The numerical labels for the sites in Fig 1 should match the abbreviated locations in the caption.

**Fig 1:**
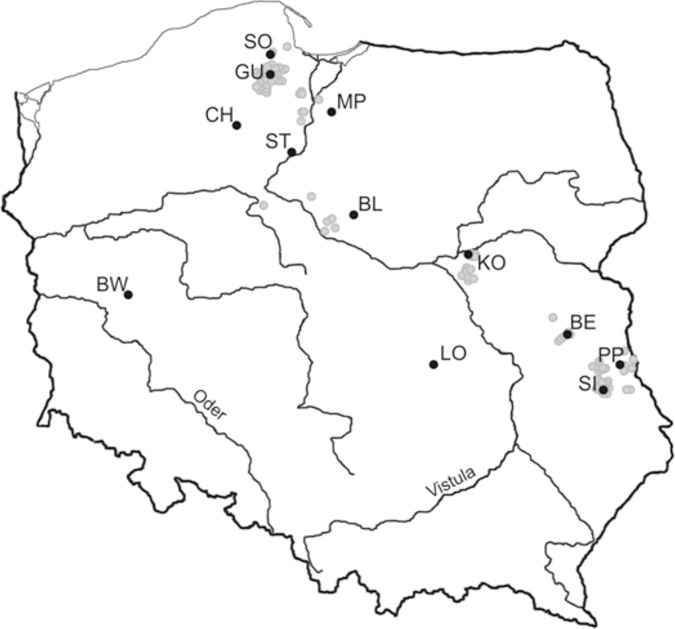
All sites of lake minnow occurrence in Poland (grey circles). Sites investigated in the present study (black circles): Sośniak (SO), Guzy (GU), Mikołajki Pomorskie (MP), Chojnice (CH), Sartowice (ST), Bledzewo (BL), Barłożnia Wolsztyńska (BW), Kowalicha (KO), Łojków (LO), Bełcząc (BE), Podpakule (PP), and Siedliszcze (SI). From each population, 48 fish were selected randomly, and samples were taken from them.

Please see the correct Fig 1 here:
